# Objective determination of peripheral edema in heart failure patients using short-wave infrared molecular chemical imaging

**DOI:** 10.1117/1.JBO.26.10.105002

**Published:** 2021-10-23

**Authors:** Aaron G. Smith, Reina Perez, Aaron Thomas, Shona Stewart, Arash Samiei, Arjun Bangalore, Heather Gomer, Marlena B. Darr, Robert C. Schweitzer, Sandhya Vasudevan, Jeffrey Cohen, J. Christopher Post, Srinivas Murali, Patrick J. Treado

**Affiliations:** a ChemImage Corporation, Pittsburgh, Pennsylvania, United States; b Allegheny General Hospital, Pittsburgh, Pennsylvania, United States

**Keywords:** molecular chemical imaging, short-wave infrared, heart failure, artificial intelligence, peripheral edema

## Abstract

**Significance**: Peripheral pitting edema is a clinician-administered measure for grading edema. Peripheral edema is graded 0, 1+, 2+, 3+, or 4+, but subjectivity is a major limitation of this technique. A pilot clinical study for short-wave infrared (SWIR) molecular chemical imaging (MCI) effectiveness as an objective, non-contact quantitative peripheral edema measure is underway.

**Aim**: We explore if SWIR MCI can differentiate populations with and without peripheral edema. Further, we evaluate the technology for correctly stratifying subjects with peripheral edema.

**Approach**: SWIR MCI of shins from healthy subjects and heart failure (HF) patients was performed. Partial least squares discriminant analysis (PLS-DA) was used to discriminate the two populations. PLS regression (PLSR) was applied to assess the ability of MCI to grade edema.

**Results**: Average spectra from edema exhibited higher water absorption than non-edema spectra. SWIR MCI differentiated healthy volunteers from a population representing all pitting edema grades with 97.1% accuracy (N=103 shins). Additionally, SWIR MCI correctly classified shin pitting edema levels in patients with 81.6% accuracy.

**Conclusions**: Our study successfully achieved the two primary endpoints. Application of SWIR MCI to monitor patients while actively receiving HF treatment is necessary to validate SWIR MCI as an HF monitoring technology.

## 1 Introduction

Heart failure (HF) can result from any structural or functional abnormality that impairs the ability of the ventricle to eject blood, referred to as systolic HF, or to fill with blood, termed diastolic HF. Prevalence of HF is widespread in the USA with 5.7 million people diagnosed; projections estimate an increase past 8 million by 2030.^1^ The economic cost of HF was estimated to be $31 billion in 2012.^1^ More than 40% of the cost is attributed to hospitalization, with volume retention and overload being the most common reason for HF hospitalization. According to a study of more than 1.3 million HF patients, 24.8% of patients were readmitted within 30 days of discharge.^2^ The American Heart Association has launched a program called Rise Above Heart Failure that assists healthcare professionals in educating patients concerning heart failure.^3^ In addition to patient education, there is a need to better understand the disease state and monitor patients both inside and outside a clinical setting in order to reduce hospital length of stay and patient readmission rate, improve quality of life for HF patients, and reduce the economic burden of HF.

HF is diagnosed based on a patient’s signs and symptoms, past medical history, family history, and test results, including blood tests and imaging. Fluid retention is one of the cardinal manifestations of HF and may accumulate in the lungs, abdomen, and extremities. Peripheral edema describes interstitial fluid volume overload in the lower extremities and is a sign of hypervolemia and progression of disease. Edema occurs when an excessive volume of fluid accumulates in the tissues, either within cells (cellular edema) or within the collagen-mucopolysaccharide matrix distributed in the interstitial spaces (interstitial edema).

The traditional clinical assessment of peripheral edema in HF patients, which is still widely used, is subjective. In this examination, a clinician applies pressure with his or her index finger to an anatomical point, usually the patient’s ankle or mid-tibia. The anatomical point is subjectively chosen to represent the location of greatest edema. This technique captures pit depth and the time needed for the skin to return to its original appearance (recovery time) as a single edema score ranging from 0 to 4+.^4^ This measurement will vary upon the experience of the clinician, time constraints, and individual patient factors.^5^ Alternative, non-invasive measurements have been investigated such as ankle circumference, but these methods lack reliability, feasibility, or correlation with the classic clinical assessment of pitting edema.^5^ Assessment of edema in the upper extremities (forearm) is not practiced clinically. Measurement of daily weight as a surrogate for volume retention is fraught with inaccuracies and is clinically unreliable.^6^

Additional methods to track patient progress including thoracic impedance measurements, pulmonary lung fluid tracking, pulse oximetry, and blood testing for natriuretic peptides are in various stages of commercial development.^7^^–^^10^ A non-invasive and non-contact monitoring option, such as imaging, would lead to reduced costs, reduced hospital readmission rate, and greater patient compliance. Invasive implantable devices, such as CardioMEMS, have shown a reduction in hospitalization rate compared to control groups.^11^ The implantable devices routinely monitor pulmonary artery pressure and edema but not peripheral edema. There is currently no commercially available imaging modality that could accurately measure and monitor peripheral edema in HF patients.

This study explores the use of short-wave infrared (SWIR) molecular chemical imaging (MCI) to non-invasively and objectively measure the level of edema in a patient’s limb. MCI has been explored for several medical applications due to the non-contact, non-destructive nature of the technique and use of non-damaging illumination that can be used to obtain multiple measurements from a given patient as needed.^12^ These characteristics are useful in medical applications in which invasive tests can lead to patient discomfort or non-compliance with physician testing schedules. SWIR MCI is potentially suitable for quantitatively measuring subcutaneous interstitial fluid due to water being a strong absorber in the SWIR spectral range. We expect to see evidence of higher water absorption in SWIR spectra from subjects with edema than from subjects without edema. Additionally, the SWIR wavelength range exhibits decreased scattering when compared with the more conventionally exploited near-infrared (NIR) range. Furthermore, the longer wavelengths in the SWIR allow deeper penetration of light into tissues.^13^^,^^14^ The penetration depth with SWIR will allow the measurement of patients with varying skin thickness, such as obese patients, and has relative insensitivity to skin pigmentation.^15^ The epidermis layer varies by body location, averaging 0.1 to 0.15 mm in thickness; the dermis layer can be 1.5 to 4 mm; and the subcutaneous layer is thicker than the dermis.^16^ The study described here serves as an opportunity to establish technical feasibility and inform future directions for the potential development of a clinical instrument. The objectives were to investigate a primary endpoint of discrimination between healthy and HF patients with edema and a second primary endpoint of whether a SWIR-based index [the CardioVerification Index (CVI)] could be conceived and used to predict the pitting edema level using the ground truth provided by the traditional pitting edema test.

## 2 Methods

### 2.1 Study Design

Patient enrollment was conducted in two cohorts, with each cohort centered at a single site. Twenty healthy volunteers were enrolled in the first cohort with no presence of edema and no other excluding criteria present, as determined by clinical personnel. Patients at Allegheny General Hospital (Pittsburgh, PA) who were under care of the principal investigator and had a documented HF diagnosis were informed of the study at an inpatient or outpatient setting. Patients were consented to the second cohort if they agreed to participate in the study. This observational study included one data collection for each enrolled patient. Forty-seven HF patients were enrolled. Data sets that did not meet the quality standards were not included, and 28 HF patients are represented in the reported results.

The edema patient population that met the quality standards was 58.3% male, 89% Caucasian, and 50% diabetic with an average age of 70.2 years. Demographics in the healthy population were closely matched in gender and ethnicity with 63% male and 95% Caucasian. The healthy population analysis pool had an average age of 39.3 years.

### 2.2 Instrument

**Fig. 1:**
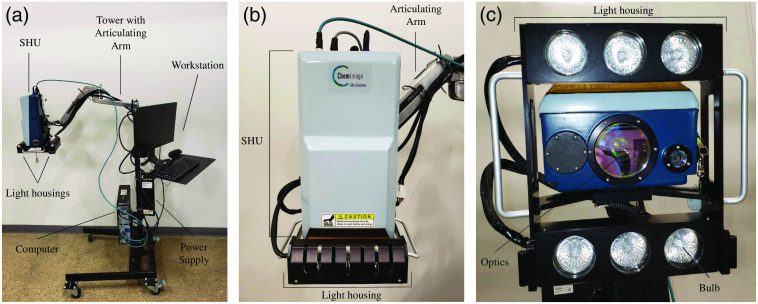
(a) Tower-based SWIR MCI instrument with main components. (b) Close-up view of the SHU attached to the articulating arm. (c) View of the lighting and optical components on the SHU.

**Fig. 2:**
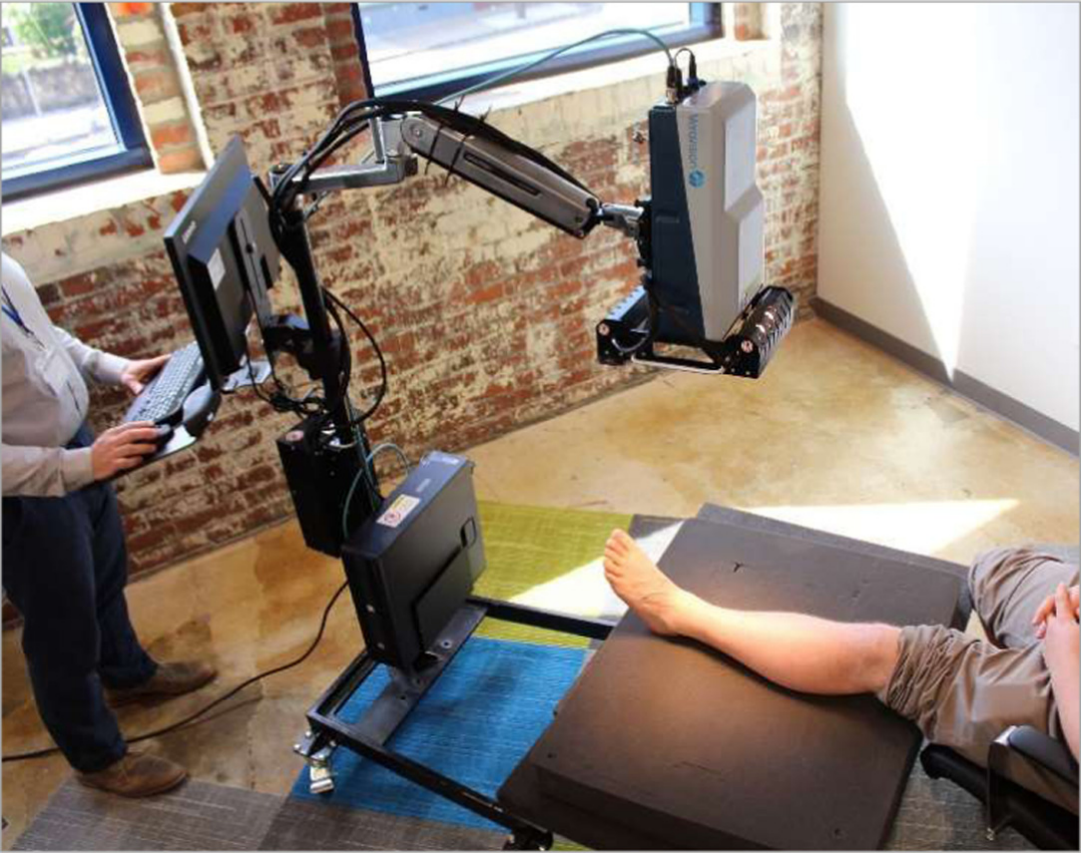
SWIR MCI of a healthy volunteer shin. Future form factors are envisioned to not require a tower and will be handheld.

A tower-based sensor was used to facilitate measurements of patients with limited or no mobility. Figure 1 shows the imaging system and major components. The sensor head unit (SHU) used in this study has been previously described in a benchtop configuration that was used to detect illegal drugs in correctional facility mail.^17^ The benchtop configuration was impractical for imaging limbs in the present study, therefore, the sensor was incorporated into the tower configuration to allow for movement of the instrument between data collection locations. Briefly, this sensor collects hyperspectral images via a SWIR multiconjugate filter (Gen 5 SWIR multiconjugate filter, ChemImage Corp., Pittsburgh, PA) that filters light, which is subsequently captured by the detector, a SWIR InGaAs focal plane array (TAU SWIR camera, FLIR, Santa Barbara, CA). This SHU is attached to an articulating arm, which enables it to be positioned over patient limbs, normal to each limb, at an approximate working distance of 70 cm (Fig. 2). A light housing has been attached to the SHU and is equipped with six 20 W halogen light bulbs (MR-16 EuroStar-Refleckto #1000010, Ushio, Tokyo, Japan) for illumination of the 163  mm×124  mm field of view. Motorized focus optics were used by the instrument operator to maintain focus between limbs and patients. Once positioned, the operator controls data collection via Spectral Kitchen^®^ software (ChemImage Corp., Pittsburgh, PA), which runs on the workstation that has been integrated onto the tower.

Daily operational qualification testing was completed to confirm that the spectral and imaging quality of the instrument was suitable for collection. Instrumentation used for this study will be useful in future studies; however, a smaller, handheld sensor will allow for increased ease of use among intended use demographics. The utility of SWIR sensors is highlighted by effective use in biological and non-biological applications.

### 2.3 Depth Penetration

Pitting edema is typically graded up to 10 mm using the conventional clinical method, so measurement of edema in deep tissue is ideal. We have modeled the noise equivalent depth of detection (NEDoD) of pitting edema using SWIR imaging, and estimate that at 1160 nm, depth of detection in normal tissue can be as deep as 9.8 mm, whereas in tissue containing edema, the depth of detection is slightly lower at 8.1 mm. In order to quantify depth sensitivity in SWIR imaging, we modeled sensitivity matrices ($\Delta R/\Delta \mu_{a}$, where R is reflectance and $\mu_{a}$ is the absorption coefficient) in healthy and edema-containing shin tissue using an optical tomography software program, NIRFAST.^18^ NEDoD values were quantified by determining a cut off depth in the sensitivity matrices with respect to the instrument noise. Realistic noise was utilized to calculate NEDoD values by extracting noise from 1 h drift measurements acquired on our instrument. Increased optical absorption was modeled in edema-containing shin tissue with ∼70% increased water content versus healthy tissue.^19^^,^^20^ Two-fold lower optical scattering was modeled in edema-containing tissue to represent dilution of the number density of scatterers in the presence of excess blood and water.^19^

### 2.4 Data Collection

Protocols and consent forms were developed by the investigators with review and approval by the Western Institutional Review Board. Prospective patients were screened against criteria defined in Table 1. Enrolled patients were assigned a deidentified study ID and metadata was collected. Pitting edema level in each shin was measured by clinical staff ∼10  min prior to measurements. Compression stockings, if applicable, were removed followed by a minimum 5-min recumbent period before data collection. Data collection involved an RGB image and a series of SWIR images covering 1000 to 1700 nm. For a given field of view, individual wavelength frames were captured at 5 nm intervals across this spectral range, with an exposure time of 32 ms. The set of wavelength frames is referred to as a hypercube, a three dimensional stack of images, where X and Y are spatial dimensions and the Z dimension represents the spectral dimension. Therefore, each pixel location in a hypercube has an individual spectrum. Collection of each hypercube takes about 50 s.

**Table 1:** Criteria used to screen patients for enrollment.

No.	Item	Criteria status
1	Males or females age 18 to 90 years	Inclusion (all cohorts)
2	Evidence from history, physical examination, imaging studies, and laboratory tests that support the diagnosis of HF	• Inclusion for HF cohort • Exclusion for healthy volunteers
3	Subjects who are incapable of providing consent may be non-compliant or whose care could be compromised	Exclusion (all cohorts)
4	Subjects who have a confirmed or suspected pregnancy	Exclusion (all cohorts)
5	Subjects with overt skin lesions	Exclusion (all cohorts)
6	Patients who have peripheral edema caused by conditions unrelated to HF	Exclusion (all cohorts)
7	Patients with a diagnosis of diabetes (type I or type II)	• Exclusion for healthy volunteers• Not an exclusion for HF cohort

**Fig. 3:**
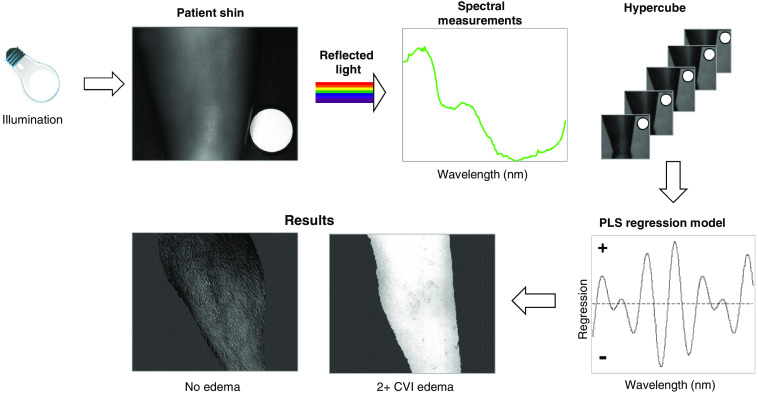
Overview of MCI data collection and processing.

Each patient had both shins and forearms imaged, and all data that met quality standards were included in the analysis. A typical data collection for all four patient limbs was 15 min or less. Data were collected using ChemImage-developed Spectral Kitchen^®^ software. Periodic reviews of enrollment were conducted by clinical staff, and de-identified metadata were shared with the sponsor. MCI technology is used for diffuse reflectance measurements where reflected light is measured from a patient’s limb, and data are recorded at specified wavelengths in a hypercube as described in Fig. 3.

### 2.5 Analysis

The data collection and analysis steps are illustrated in Fig. 3. The data collection starts with the illumination of the patient’s shin or forearm. A molecular chemical image or hypercube, comprising an image frame at each wavelength interrogated is subsequently collected. Spectra corresponding to soft tissue are extracted from the hypercubes and applied to multivariate statistical and classification methods, such as partial least squares regression (PLSR). The PLSR model generates, from the spectra, regression vectors that are used to predict CVI values for any given spectrum (pixel). The two output images (“no edema” and “2+ CVI edema”) illustrate the fact that the PLSR model can be used to calculate CVI values for every spectrum (pixel) in an input hypercube, thereby outputting a PLS score image.

#### 2.5.1 Preprocessing

The impact of non-uniform illumination was reduced by dividing patient hypercubes by an image of a 99% reflectance standard (Gigahertz-Optik, Amesbury, MA) filling the field of view. Instrument response was additionally accounted for by performing a local background division of a reflectance standard placed in the field of view with the patient limb. Processing steps were carried out using ChemImage Xpert^®^ software (ChemImage, Corp., Pittsburgh, PA).

#### 2.5.2 Data extraction

Ten average spectra were extracted from SWIR hypercubes by selecting regions of interest (ROIs) comprising 462 pixels per ROI. In the shin hypercubes, five ROIs on either side of the tibia were sampled. The tibia was avoided because the bone is more reflective than the soft tissue and thereby distorts the signal associated with edema. Forearm average spectra were sampled with five ROIs on each side of the limb. The spectral extraction was enabled by a graphical tool in ChemImage Xpert that extracts the average spectrum associated with a given ROI drawn by a user on an image frame associated with a hypercube in the GUI. The software supports exporting the spectra to a spectral file that can then be used in downstream processing.

#### 2.5.3 Supervised classification models for discrimination analysis to support endpoint 1

Partial least squares discriminant analysis (PLS-DA) is a statistical method of analysis employed for data reduction, model creation, and data classification.^21^^–^^23^ This supervised classification method was performed in MATLAB with cross validation and was used to create a two-class model discriminating between spectra from healthy volunteers and those from HF patients exhibiting peripheral edema. PLS-DA is a binary classification method and the results presented consider shins and forearms to be separate populations for analysis. All shins are used in the development of the shin model. However, leave-one-out cross validation is used so the prediction of CVI values for a given shin does not include the spectra in that shin. In essence, a separate model is generated and used for each patient shin. The same process is followed for the forearm models.

The performance of a PLS-DA model is assessed through a receiver operating characteristic (ROC) curve analysis. A ROC curve is a plot showing sensitivity versus 1-specificity of a test for a binary system. The area under the ROC curve (AUC) is a measure that is often used to compare the performance of ROC curves using a single value. An AUC of 1 indicates 100% classification accuracy of data.

#### 2.5.4 Supervised quantitative analysis to support endpoint 2

PLSR is closely related to PLS-DA and was also performed in MATLAB. Both algorithms share the same PLS approach that reduces the dimensionality of the data space from n dimensions (the number of wavelengths) to k dimensions (the number of selected basis vectors) in a manner that preserves the maximum amount of variance from both the X-block (spectral data) and the Y-block (edema score information).^24^ PLSR, like PLS-DA, is a supervised method that requires a set of training data consisting of spectra and edema scores to result in a mathematical model that allows the prediction of edema scores for data not included in the training process. PLSR is a regression method that generates real values corresponding to non-integer assignments of edema scores. The values obtained from PLSR analysis are termed the CVI, which are compared to the pitting edema ground truth by rounding a CVI score to the nearest whole integer.

The PLSR approach used in this work utilizes a hierarchy of models. The first model discriminates between pitting edema levels of 0 (no evidence of edema) and all other levels of pitting edema. The second model discriminates between edema levels of 1+ versus the combination of 2+, 3+, and 4+. The third model discriminates 4+ from a combination of 2+ and 3+. The final model discriminates between pitting edema levels of 2+ and 3+. The forearm population available for this study did not have 4+ data sets, so the third model was not needed for that population. The PLSR models were built using cross validation and were evaluated using accuracy as the figure of merit.

## 3 Results

**Fig. 4:**
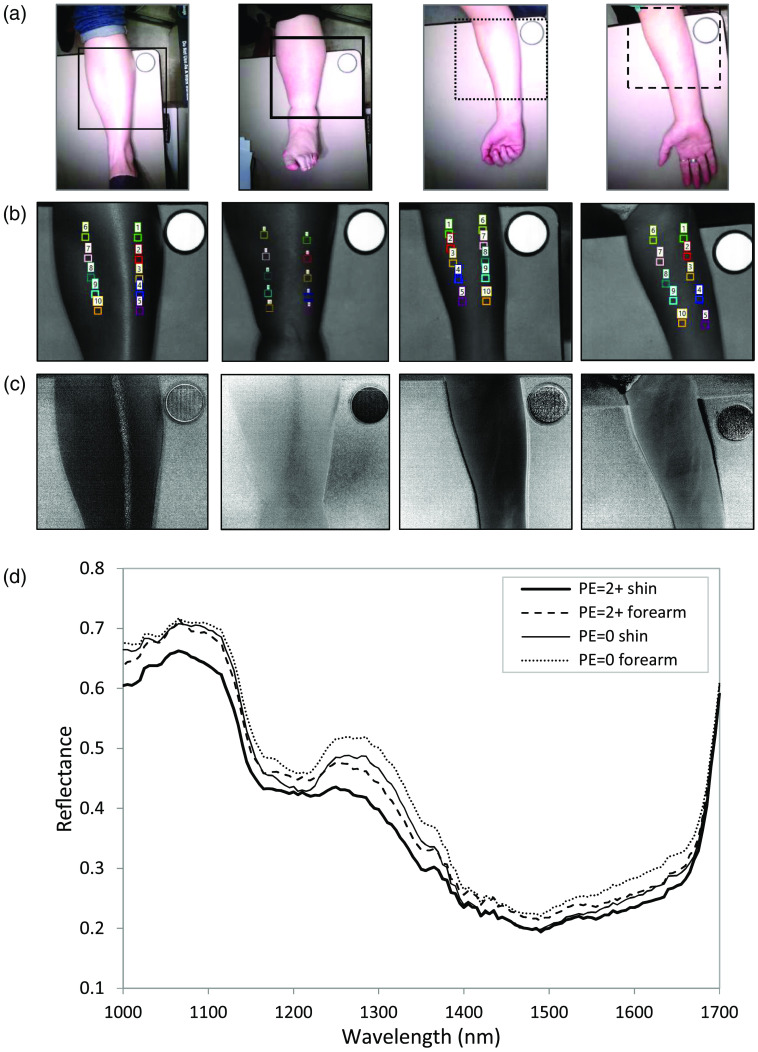
Example data from a healthy volunteer and an edema patient. (a) RGBs of a healthy PE=0 volunteer shin (left), 2+ edema shin (middle left), healthy PE=0 volunteer forearm (middle right) and a 2+ edema forearm (right). Areas sampled for SWIR analysis indicated by blue boundaries. (b) Spectral images at 1200 nm and ROIs were used for spectral extraction. (c) Processed images displaying bone and soft tissue regions in shin and vasculature in forearm images. (d) Average spectra from the ROIs.

Figure 4(a) displays example shin and forearm RGB images from a patient with edema and a volunteer without edema, whereas Fig. 4(b) shows a spectral image at 1200 nm indicating ROIs from which spectra were sampled. The images in Fig. 4(b) are displayed at a consistent wavelength between limbs using 1200 nm; however, any of the evaluated SWIR wavelengths could be used to illustrate the data. Figure 4(c) shows example processed images that may reveal features, such as bone or vascular locations not obvious in RGB or raw spectral images. Spectra were extracted from soft tissue regions to eliminate influence from the tibia, which is more reflective. The extracted regions represented several areas of the shin to account for local edema changes and variability of molecular species. Figure 4(d) displays representative reflectance spectra from the ROIs for both shins and forearms.

The principal macromolecules represented in the SWIR spectra are water, lipids, and collagen.^25^ Lipids, and to a lesser extent, collagen and water, absorb between ∼1120 and 1230 nm.^25^^,^^26^ The peaks in this spectral region are associated with the second overtone of C─H stretching in lipids and collagen and a vibrational overtone of the O–H bond in water. The larger envelope from 1300 to 1650 nm encompasses mostly collagen and water, peaking at ∼1480  nm, and some lipid, absorbing closer to 1400 nm.^25^^,^^26^ The presence of collagen is reflected in the absorption peaking at ∼1500  nm, representing a combination of ${\mathrm{CH}}_{2}$ stretching and non-stretching modes. Water and lipids are represented by the first overtone of OH stretching, closer to 1430 nm.

A portion of collected data was not suitable for analysis due to incorrect wavelength step size (wavelength distance between collected images in a hypercube), file save failures, file corruptions, or protocol issues such as the absence of the flatfielding correction standard (99% reflectance standard disc) or incorrect standoff distance. In total, 27 of 94 shins were removed from the HF population, corresponding to 11 subjects in which both shins were excluded, and 5 subjects in which one shin was excluded. Similarly, 12 forearm data sets were found to be not evaluable. The removal of data due to quality included one volunteer in the healthy population who had an edema level other than 0 and another with a diagnosis of diabetes.

The primary analysis endpoints for the study were to (1) resolve if SWIR MCI can accurately discriminate between healthy and edema patients and (2) investigate if SWIR MCI can accurately discriminate amongst all pitting edema levels. Acceptance criteria for each endpoint were established as an accuracy of 80% or greater. Two-class PLS-DA models were built to discriminate between healthy patients without pitting edema and HF patients with all levels of pitting edema. The distribution of edema in evaluable limbs is summarized in Table 2. Pitting edema ground truth level was measured in the shin area as part of the routine care of HF patients. Forearm pitting edema was not collected as part of this pilot study, and the applicable shin ground truth was applied to the forearm data. The forearm population was selected using corresponding forearm data from the final shin population. Some data sets from the forearm population did not meet the data quality requirements outlined above and were removed from the evaluated data.

**Table 2:** Distribution of edema in patients.

No.	Pitting edema level	Number of evaluable shins	Number of evaluable forearms
1	0/absence of edema	36	36
2	1+	10	8
3	2+	31	27
4	3+	22	20
5	4+	4	0

**Fig. 5:**
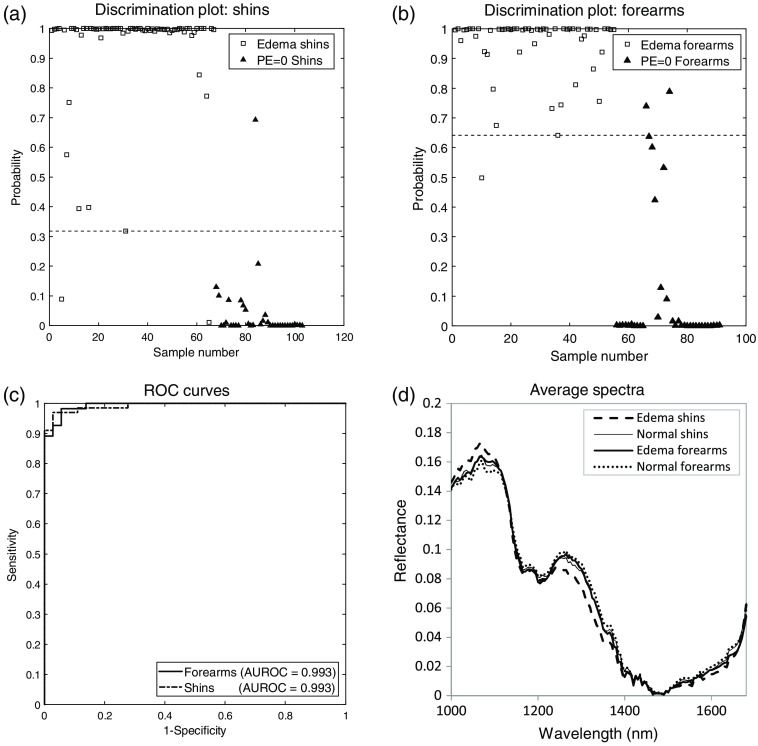
Differentiation of healthy and edema populations using PLS-DA. (a) Discrimination plot for shin data. Shin data are classified as edema for values above the dotted threshold. (b) Discrimination plot for forearm data. Forearm data are classified as edema for values above the dotted threshold. (c) ROC curves for two class PLS-DA models discriminating healthy patients from HF patients with peripheral edema. (d) Average reflectance spectra for edema and healthy shins and forearms used in models.

The discrimination plots for the shin and forearm models are shown in Figs. 5(a) and 5(b). The ROC curves, shown in Fig. 5(c), correspond to an AUC of 0.993, accuracy of 97.1%, sensitivity of 97.0%, and specificity of 97.2% for the shin population. The test for forearm edema had an AUC of 0.993, accuracy of 96.7%, sensitivity of 98.2%, and specificity of 94.4%. Figure 5(d) displays the average spectra from the edema and healthy population shins and forearms. These results are encouraging, and suggested additional analysis be undertaken to understand if discrimination between levels of pitting edema is feasible.

**Fig. 6:**
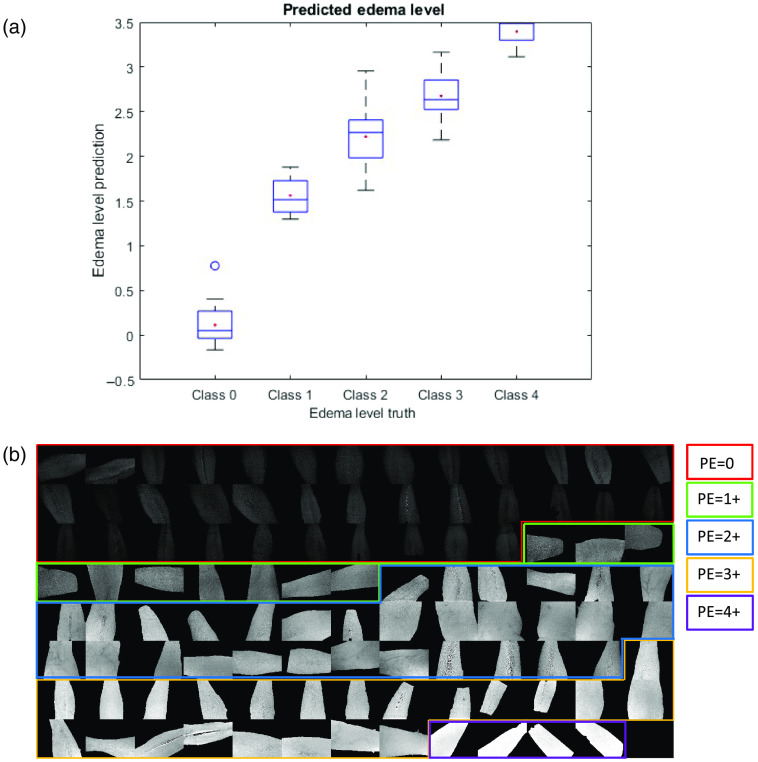
(a) Box plot for hierarchical models comparing the predicted shin edema level using CVI scores and ground truth provided by clinical staff. (b) Output images for the hierarchical PLSR model. Shins are displayed in row-order of increasing ground truth edema level. Shins 1 to 36 starting in upper left corner (PE=0 ground truth; red box); shins 37 to 46 (PE=1+ ground truth; green box); shins 47 to 60 (PE=2+ ground truth; blue box); shins 61 to 78 (PE=3+ ground truth; yellow box); and shins 79 to 80 (PE=4+ ground truth; purple box). The overall classification accuracy for this model is 86%.

**Fig. 7:**
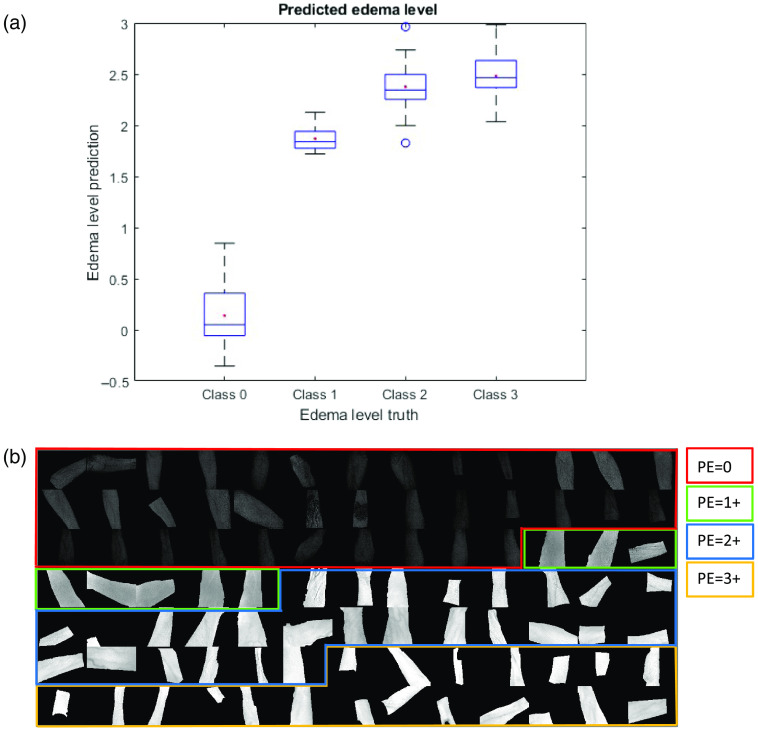
(a) Box plots for hierarchical models comparing the predicted edema level using CVI scores and ground truth provided by clinical staff. (b) Example output images for the hierarchical PLSR model. Forearms are displayed in row-order of increasing ground truth edema level. Forearms 1 to 36 starting in upper left corner (PE=0 ground truth; red box); forearms 37 to 46 (PE=1+ ground truth; green box); forearms 47 to 58 (PE=2+ ground truth; blue box); forearms 59 to 76 (PE=3+ ground truth; yellow box). The overall classification accuracy for this model is 71.1%.

The second primary endpoint was investigated using hierarchical PLSR to build a model using extracted spectra and the ground truth edema level as the dependent variable. The shin model generated CVI scores that predict healthy and HF patient edema levels with an accuracy of 81.6% when compared to the ground truth. This model was then applied to shin hypercubes to generate score images that enabled visualization of edema in participant shins. Figure 6(a) shows box plots of predicted pitting edema level versus the provided ground truth with the standard deviation represented as a box. The red dot indicates the median, the center line in the box is the mean, and the bottom and top edges of the box represent the 25th and 75th percentiles, respectively. The “whiskers” around the box plot represent the extremes of the distribution that are not considered outliers and the outliers are represented by open and closed circles, where open circles are outliers and closed circles are extreme outliers. Figure 6(b) comprises the PLSR score images generated when this model is applied to subject hypercubes. Each pixel in the image is generated by multiplying the spectrum for that pixel by the regression vector that is the output of the PLSR model building exercise. By processing every pixel, a score image for that model is assembled. The average intensity of the pixels, in general, increases as the predicted edema level increases. The same methodology was applied to the forearm population as shown in Fig. 7. A hierarchical PLSR model using forearm data discriminated edema levels with an accuracy of 63.8%.

## 4 Discussion

In this pilot study, the primary endpoint of a non-invasive and non-contact SWIR MCI-based approach to discriminate between limbs with and without edema was achieved as judged by the high accuracy of the two-class PLS-DA model. The second primary analysis endpoint of predicting edema level in patients gave encouraging results, but a larger study is needed to obtain and train on greater populations for all edema levels. The majority of biological tissue-based spectroscopic studies have focused on the measurement of chromophores such as hemoglobin, which absorb in the visible wavelength range.^27^^–^^29^ However, the SWIR region offers benefits that have not been exploited in high volume until more recently, due to recent developments in SWIR-based optics, e.g., InGaAs sensors.^15^ First, the SWIR spectral region is highly favorable for thicker samples due to the longer wavelengths and transparency of the region to blood. Additionally, the absorption coefficients of key chromophores: water, lipids, and collagen are higher in the SWIR region compared with the visible and NIR regions. For example, the absorption coefficient of water is more than 60 times higher at 1440 nm than at the 975-nm NIR peak.^30^ In a similar trend, lipid absorbs many magnitudes more at 1210 nm than at 930 nm.^28^^,^^29^

**Fig. 8:**
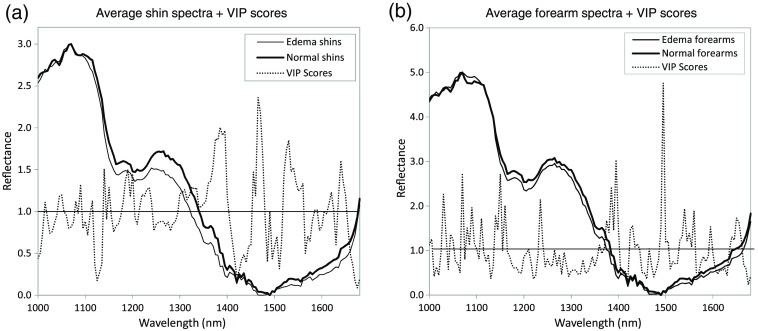
(a) Average shin reflectance spectra and VIP scores calculated from the PLS-DA model for edema in shins. (b) Average forearm reflectance spectra and VIP scores calculated from the PLS-DA model for edema in forearms.

The basis of discrimination among the populations can be explored by comparing spectra from the healthy and edema populations. Figures 8(a) and 8(b) show average normal and edema reflectance spectra representing the shin and forearm data, respectively, used to generate the two PLS-DA models. In the key region of 1120 to 1230 nm, it is clear that absorption is higher in spectra from subjects with edema in both shins and forearms. We can exploit spectral analysis further by evaluating the variable importance in projection (VIP) scores from the model. Shin and forearm VIP scores are shown alongside average model spectra in Figs. 8(a) and 8(b), respectively. A VIP score is a measure of a variable’s importance in a PLS model. VIP scores can be used for variable selection and for identifying the spectral regions of importance in a model.^31^ A variable (in this case, wavelength) with a VIP score >1 can be considered important in a given model. The VIP scores associated with the PLS-DA shin model indicate the spectral regions that play a significant part in discriminating between healthy subjects and those with edema, regardless of grade. Several spectral locations with VIP scores >1 are evident. As expected, these locations represent all three macromolecules—lipids, collagen, and water. VIP scores higher than 1 at 1140 and 1150 nm reflect water content; 1200 and 1235 nm correspond to lipid and collagen absorption. However, the highest VIP scores are at 1385, 1465, and 1530 nm. This broad spectral envelope corresponds to the absorption of mostly water and collagen, and lipids to a lesser extent. At 1385 and 1465 nm, OH stretching likely causes the observed absorption, and at 1530 nm, we expect there to be water influence as well as contribution from ${\mathrm{CH}}_{2}$ stretching and non-stretching vibrations in collagen. The forearm VIP scores are very similar to the shin VIP scores.

What these analyses tell us is that not only are edema and normal shins and forearms distinguishable using SWIR MCI, the basis of discrimination is water content in addition to collagen and lipid differences. Because subjects with high levels of edema can have as high as a 70% increase in water content,^19^^,^^20^ we estimate that water differences impart a high bearing on differentiating between edema and normal tissue spectra.

Among other collected metadata, the influence of diet and medications will be monitored to determine effects on results during treatment. Alternate prediction strategies, such as artificial intelligence (AI), may be employed when a larger data set is available with greater patient populations. Currently, PLSR is a good choice for model building, given the limited and the spectral nature of the data. Deep neural networks are often chosen for machine learning and AI but are a better choice when the data consists of large training sets of images. Certain AI tools, such as data augmentation, will be investigated via spectral additions of water to current samples to increase the training set coverage and provide more robust models. Greatly increased data collections for an individual patient when used in a monitoring mode could allow for adaptive background estimation which could better model the spectral background. Next generation handheld sensors will incorporate more advanced computer vision techniques for standoff distance and angle calculations as well as for motion correction.

The prediction accuracy of the shin model is greater than that of the forearm model. The ground truth for a given shin was used as ground truth for the corresponding forearm. This may have influenced the degradation of forearm accuracy, as shin edema may be more severe due to gravity. The effect of sampling location will continue to be investigated, as flexibility in this area will increase clinical utility for patients with physical impairments, morbidly obese populations, and limited mobility patients. Evaluating the forearm, for example, which is not currently used to measure pitting edema, allows us to assess the upper extremity as an option for edema measurement, which would give us flexibility when designing future form factors.

Subsequent clinical trials will, when possible, include a more diverse population in terms of ethnicity, pitting edema level, and age to better capture biological variability and increase the robustness of models.

In an additional experiment, individual two-class PLS-DA models were built from subjects’ right and left shin measurements. Using individual shin pitting edema ground truth, the accuracy of the right shin model was higher than that of the left side shin model (100% versus 96.1%, respectively; data not shown). Although not currently statistically different, we will continue to monitor this trend in future clinical trials to understand if sampling from multiple limbs is necessary, and to understand optimal sampling location. Any metadata in a patient’s history that may influence the results, such as a heart bypass operation or which veins were harvested from a patient’s leg for bypass surgery, will be monitored.

The feasibility of monitoring dynamic peripheral edema levels in patients over time, including patients who are receiving treatment, will be investigated in upcoming clinical trials. Ultimately, these trials will help understand if MCI technology used consistently on a periodic basis can reduce readmission rates for HF patients. Efforts are underway to develop a hand-held form factor that will automate features where possible to reduce the training burden. These efforts are being conducted by CardioVere, a spinout of ChemImage. The eventual goal is to develop strategies to automatically integrate MCI results into the electronic health record to alert clinicians when a patient has a condition change and requires clinical intervention.

We acknowledge some of the limitations of the study, including underrepresentation of non-Caucasian ethnicities and patients with 4+ pitting edema. Data sets from four enrolled non-Caucasian patients met data quality standards and were included in the reported models. Although we currently do not have evidence suggesting that a change in performance would occur when including a larger number of non-Caucasian patients, this will be investigated in future studies with a larger patient population. Though we only included HF patients with pitting edema, it is possible that some of the patients had non-pitting edema, which may have influenced the results. The cohort without HF had a lower average age than the cohort with HF. Skin thickness may decrease with increased age and this will be considered in future protocols. Finally, this study included forearm assessment for edema, which is typically not part of routine evaluation of HF patients. Understanding the body locations useful in the evaluation of edema will be explored in additional studies. Although this study only obtained ground truth from the shin area, the protocols for future studies are anticipated to collect ground truth from each location sampled. Importantly, the evaluation of sampling locations is not limited only to the extremities used in this study.

## 5 Clinical Perspectives

Tracking volume status in HF patient care is vital to prevent the progression of the disease. Accurate tracking helps to manage symptoms better by making simple lifestyle changes and modifying therapeutics. This technology may provide a reliable, objective, non-invasive, and non-contact methodology for quantitative peripheral edema measurement. As the technology matures, it is envisioned that patient self-monitoring, with wireless transmission of edema levels to physicians from home, can aid clinicians in monitoring HF patients for improved patient outcomes and to improve the future of individualized medicine. This increases the chance of modifying treatment plans appropriately based on accurate monitoring of edema level. This ultimately may reduce HF hospital readmission rates and unscheduled, emergent visits to the clinic or emergency room.

## References

[1] Mozaffarian D. et al., “Heart disease and stroke statistics - 2016 update: a report from the American Heart Association,” Circulation 133(4), e38-360 (2016). CIRCAZ 0009-7322 10.1161/CIR.0000000000000350 26673558

[2] Cheng R. et al., “Outcomes in patients with heart failure with preserved, borderline, and reduced ejection fraction in the Medicare population,” Am. Heart J. 168(5), 721–730 (2014). 10.1016/j.ahj.2014.07.008 25440801

[3] American Heart Association (editorial staff), “Rise above heart failure toolkit for healthcare professionals,” AHA, https://www.heart.org/en/health-topics/heart-failure/heart-failure-tools-resources/rise-above-heart-failure-toolkit (accessed 4 May 2020).

[4] “Grading of edema,” Med-Health, http://www.med-health.net/Edema-Grading.html (accessed 4 May 2020).

[5] Brodovicz K. et al., “Reliability and feasibility of methods to quantitatively assess peripheral edema,” Clin. Med. Res. 7(1–2), 21–31 (2009). 10.3121/cmr.2009.819 19251582 PMC2705274

[6] Lynga P. et al., “Weight monitoring in patients with severe heart failure (WISH). A randomized controlled trial,” Eur. J. Heart Fail 14(4), 438–444 (2012). 10.1093/eurjhf/hfs023 22371525

[7] Sato M. et al., “Bioelectrical impedance analysis in the management of heart failure in adult patients with congenital heart disease,” Congenit. Heart Dis. 14(2), 167–175 (2019). 10.1111/chd.12683 30351489

[r8] Brunner-La Rocca H. Sanders-van Wijk S., “Natriuretic peptides in chronic heart failure,” Card Fail Rev. 5(1), 44–49 (2019). 10.15420/cfr.2018.26.1 30847245 PMC6396059

[r9] Watanabe E. et al., “Prognostic importance of novel oxygen desaturation metrics in patients with heart failure and central sleep apnea,” J. Card Fail. 23(2), 131–137 (2017). 10.1016/j.cardfail.2016.09.004 27615064 PMC5276717

[10] Uriel N. et al, “Relationship between noninvasive assessment of lung fluid volume and invasively measured cardiac hemodynamics,” J. Am. Heart Assoc. 7(22), e009175 (2018). 10.1161/JAHA.118.009175 30571493 PMC6404458

[11] Givertz M. et al., “Pulmonary artery pressure-guided management of patients with heart failure and reduced ejection fraction,” J. Am. Coll. Cardiol. 70(15), 1875–1886 (2017). JACCDI 0735-1097 10.1016/j.jacc.2017.08.010 28982501

[12] Lu G. Fei B., “Medical hyperspectral imaging: a review,” J. Biomed. Opt. 19(1), 010901 (2014). JBOPFO 1083-3668 10.1117/1.JBO.19.1.010901 PMC3895860 24441941

[13] Sordillo D. C. et al., “Short wavelength infrared optical windows for evaluation of benign and malignant tissues,” J. Biomed. Opt. 22(4), 045002 (2017). 10.1117/1.JBO.22.4.045002 28384701

[14] Thimsen E. Sadtler B. Berezin M. Y., “Shortwave-infrared (SWIR) emitters for biological imaging: a review of challenges and opportunities,” Nanophotonics 6(5), 1043–1054 (2017). 10.1515/nanoph-2017-0039

[15] Zhang H. et al., “Penetration depth of photons in biological tissues from hyperspectral imaging in shortwave infrared in transmission and reflection geometries,” J. Biomed. Opt. 21(12), 126006 (2016). JBOPFO 1083-3668 10.1117/1.JBO.21.12.126006 27930773 PMC5147011

[16] Kolarsick P. Kolarsick M. Goodwin C., “Anatomy and physiology of the skin,” J. Deramtol. Nurses Assoc. 3(4), 203–213 (2011). 10.1097/JDN.0b013e3182274a98

[17] Schweitzer R. et al., “Automated chemical imaging identification of illegal drugs in correctional facilities mail,” J. Chemometr. 32(10), e3038 (2018). JOCHEU 0886-9383 10.1002/cem.3038

[18] Dehghani H. et al., “Near infrared optical tomography using NIRFAST: algorithm for numerical model and image reconstruction,” Commun. Numer. Methods Eng. 25(6), 711–732 (2008). CANMER 0748-8025 10.1002/cnm.1162 20182646 PMC2826796

[19] Weber J. R. et al., “Multispectral imaging of tissue absorption and scattering using spatial frequency domain imaging and a computed-tomography imaging spectrometer,” J. Biomed. Opt. 16(1), 011015 (2011). 10.1117/1.3528628 21280902 PMC3055588

[20] Lin A. J. et al., “Spatial frequency domain imaging of intrinsic optical property contrast in a mouse model of Alzheimer’s disease,” Ann. Biomed. Eng. 39(4), 1349–1357 (2011). ABMECF 0090-6964 10.1007/s10439-011-0269-6 21331663 PMC3069335

[21] Martens H. Naes T., Multivariate Calibration, John Wiley & Sons, New York (1992).

[r22] Barker M. Rayens W., “Partial least squares for discrimination,” J. Chemom. 17(3), 166–173 (2003). 10.1002/cem.785

[23] Stewart S. et al., “Distinguishing between renal oncocytoma and chromophobe renal cell carcinoma using Raman molecular imaging,” J. Raman Spectrosc. 45(4), 274–280 (2014). 10.1002/jrs.4460

[24] Beebe K. Pell R. Seasholtz S., Chemometrics: A Practical Guide, 1st ed., pp. 286–287, John Wiley & Sons, New York (1998).

[25] Wilson R. H. et al., “Review of short-wave infrared spectroscopy and imaging methods for biological tissue characterization,” J. Biomed. Opt. 20(3), 030901 (2015). JBOPFO 1083-3668 10.1117/1.JBO.20.3.030901 25803186 PMC4370890

[26] Allen T. J. et al., “Spectroscopic photoacoustic imaging of lipid-rich plaques in the human aorta in the 740 to 1400 nm wavelength range,” J. Biomed. Opt. 17(6), 061209 (2012). JBOPFO 1083-3668 10.1117/1.JBO.17.6.061209 22734739

[27] Zuzak K. J. et al., “Visible reflectance hyperspectral imaging: characterization of a noninvasive, in vivo system for determining tissue perfusion,” Anal. Chem. 74, 2021–2028 (2002). ANCHAM 0003-2700 10.1021/ac011275f 12033302

[r28] Nachabe R. et al., “Diagnosis of breast cancer using diffuse optical spectroscopy from 500 to 1600 nm: comparison of classification methods,” J. Biomed. Opt. 16(8), 087010 (2011). JBOPFO 1083-3668 10.1117/1.3611010 21895337

[29] Tsai C. Chen J. Wang W., “Near-infrared absorption property of biological soft tissue constituents,” J. Med. Biol. Eng. 21, 7–14 (2001). IYSEAK 0021-3292

[30] Hale G. M. Querry M. R., “Optical constants of water in the 200-nm to 200-m wavelength region,” Appl. Opt. 12(3), 555–563 (1973). APOPAI 0003-6935 10.1364/AO.12.000555 20125343

[31] Chong I. Jun C., “Performance of some variable selection methods when multicollinearity is present,” Chemometr. Intell. Lab Syst. 78(1–2), 103–112 (2014). 10.1016/j.chemolab.2004.12.011

